# A Survey on Recent Advances in Machine Learning Based Sleep Apnea Detection Systems

**DOI:** 10.3390/healthcare9070914

**Published:** 2021-07-20

**Authors:** Anita Ramachandran, Anupama Karuppiah

**Affiliations:** 1 Department of Computer Science & Information Systems, BITS, Pilani 560001, India; 2 Department of Electrical & Electronics Engineering, BITS, Pilani-K K Birla Goa Campus, Near NH17B, Zuari Nagar, Sancoale 403726, India; anupkr@goa.bits-pilani.ac.in

**Keywords:** sleep apnea, machine learning, deep learning, wearable systems

## Abstract

Sleep apnea is a sleep disorder that affects a large population. This disorder can cause or augment the exposure to cardiovascular dysfunction, stroke, diabetes, and poor productivity. The polysomnography (PSG) test, which is the gold standard for sleep apnea detection, is expensive, inconvenient, and unavailable to the population at large. This calls for more friendly and accessible solutions for diagnosing sleep apnea. In this paper, we examine how sleep apnea is detected clinically, and how a combination of advances in embedded systems and machine learning can help make its diagnosis easier, more affordable, and accessible. We present the relevance of machine learning in sleep apnea detection, and a study of the recent advances in the aforementioned area. The review covers research based on machine learning, deep learning, and sensor fusion, and focuses on the following facets of sleep apnea detection: (i) type of sensors used for data collection, (ii) feature engineering approaches applied on the data (iii) classifiers used for sleep apnea detection/classification. We also analyze the challenges in the design of sleep apnea detection systems, based on the literature survey.

## 1. Introduction

Sleep apnea is a sleep disorder in which a sleeping person’s breathing is disturbed. It is prevalent in adults as well as a small percentage of the juvenile population [1]. Subjects suffering from sleep apnea undergo periods of no or shallow breathing during their sleep. The former condition in which breathing stops temporarily is referred to as apnea, while the latter condition of periods of shallow breathing or airflow reduction is called hypopnea. Clinical comorbidities can result from either condition and, therefore, both are detrimental to a person’s well-being [2]. The physiological symptoms of sleep apnea include snoring, gasping for air during sleep, waking up with dry mouth and, in general, low sleep quality, thereby leading to low attention, insomnia, decrease in cognitive skills, accidents, memory loss and depression. In addition to the low quality of life caused by sleep deprivation and fatigue, sleep apnea may also lead to severe issues such as diabetes, cardiovascular problems, hypertension, neurological issues, and liver problems. Due to the global prevalence of sleep apnea as well as the direct and indirect long-term problems it brings about, it is important to diagnose and treat this condition. In this paper, we review the recent state-of-the-art research in the application of machine learning for sleep apnea detection. The review covers the parameters and sensors used, and feature engineering approaches for enabling sleep apnea detection using machine learning.

There are three types of sleep apnea:

- Obstructive sleep apnea (OSA) occurs due to improper functioning of the upper respiratory tract. When the muscles of the hard palate in the back of the throat that supports that soft palate relax, the soft palate blocks the passage of air to the respiratory system. This leads to stoppage of breathing for short durations [3].
- Central sleep apnea (CSA) occurs when the brain fails to generate or transmit signals that control breathing muscles. This leads to short durations of time when the subject does not breathe at all.
- Complex sleep apnea syndrome is manifested with central apnea persisting even after obstructive events have disappeared with PAP therapy [4].

**Figure 1:**
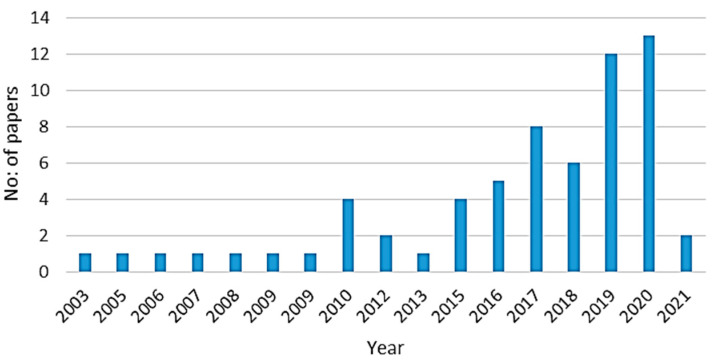
Year-wise Distribution of Papers.

Javaheri et al. [3] describe the etiological risk factors for sleep apnea and its consequences. In this paper, we describe the recent research in the application of machine learning for sleep apnea detection. Figure 1 presents the distribution of the number of papers selected for this study from 2003 through 2021. The technical focus of this study includes the following facets of sleep apnea detection: (i) type of sensors used for data collection, (ii) feature engineering approaches applied on the data, and (iii) classifiers used for sleep apnea detection/classification.

This paper is organized as follows: In Section 2, we briefly explain how sleep apnea is diagnosed, and the biomedical parameters along with their derivatives that aid in the process. Subsequently, we examine the drawbacks of the standard tests for sleep apnea detection, and reason the need for leveraging on the advances in machine learning and wearable device technologies for the same. Section 3 details the recent studies on intelligent sleep apnea detection mechanisms using classic machine learning and deep learning based solutions, using single markers as well as sensor/feature fusion. Section 4 outlines the recent studies in sleep apnea detection using machine learning on data generated by environmental sensors and the significance of including features related health profiles, during classifier training. We conclude our paper with our observations on the various factors that influence the performance of machine learning classifiers for sleep apnea detection.

## 2. Background

### 2.1. Diagnosis of Sleep Apnea

Clinical manifestations of sleep apnea conditions include variations in oxygen saturation levels, respiratory effort, and heart rate. Gottlieb et al. [5] describes the pathophysiology, assessment and treatment of obstructive sleep apnea. The PSG test is the gold standard in the diagnosis of this condition [1]. This test is conducted in dedicated sleep labs under the supervision of trained personnel. It is time consuming, and requires subjects to be connected to instruments measuring various biomedical and physiological parameters. The test monitors upper airway flow, respiratory effort, and biomedical and physiological parameters such as electroencephalogram (EEG), electrocardiogram (ECG), and oxygen saturation (SPO2) [1]. EEG helps detect electrical activity in the brain and related disorders. This is measured using an EEG machine. ECG analyzes the rhythm of heartbeats and blood flow to the heart muscles and is measured using an ECG machine or a single lead ECG. SPO2 indicates the measure of oxygen in the blood. A pulse oximeter is used to measure SPO2. In addition, thoracic and abdominal signals as well as acoustic signals generated by respiratory effort or snoring can also aid in the detection of sleep apnea.

Various parameters useful in the diagnosis of sleep apnea can be derived from the above-mentioned signals. Analysis of ECG yields Heart Rate Variability (HRV), ECG derived respiration (EDR), Cardiopulmonary coupling (CPC), and Ballistocardiography (BCG) parameters.

- HRV measures the variation in the time interval between consecutive heartbeats, known as the R-R interval. Previous research shows that variation in R-R interval is a symptom of apneic events, and hence can provide the physiological basis of using R-R series to detect OSA. Analyzing HRV, however, poses certain challenges. This includes special attention to signal quality and elimination of background noise, along with using a sensitive R-wave detection algorithm. Furthermore, interpretation of HRV is difficult in patients who have atrial fibrillation or those with irregular heartbeats [6].
- Instantaneous Heart Rate (IHR) is the number of times the heart would beat if successive R-R intervals were constant.
- EDR measures respiratory activity from ECG. An explanation of the relation between EDR and ECG is given in [7]. The respiratory effort causes changes in the position of the ECG electrodes, which in turn affects the amplitude of the ECG signals. EDR is the surrogate respiration signal derived from the amplitude variations of the ECG signals. There are several techniques to derive EDR from ECG [8].
- CPC quantifies the degree of coherent coupling between HRV and variations of the R-wave amplitude caused by modulation of the respiratory tidal volume. CPC can be of high or low frequency coupling (HFC, LFC); the former is indicative of stable sleep, while the latter is associated with sleep instability. A special characteristic of LFC, so-called elevated LFC, can be used to detect periods of apnea and hypopnea [9].
- Ballistocardiography (BCG) is a noninvasive method based on the measurement of body motion (body movements such as displacement, velocity, and acceleration), generated by the ejection of blood by the heart, at each cardiac cycle. This is measured using devices that can measure the body recoil force produced as a result of ejection of blood [10].
- A parameter that may be related to HRV is Pulse Rate Variability (PRV), which is measured from photoplethysmography (PPG) sensors [11]. PPG sensors use a light source and a photodetector on the skin to characterize blood circulation.

Oxygen Desaturation Index (ODI) is a metric derived from SPO2, which represents the number of times the oxygen level in blood falls for more than 10 s, divided by the number of sleep hours. ODI is defined as the number of times that oxygen desaturation was ≥3% per hour of sleep [12].

The above mentioned parameters are used to infer certain measures to ascertain the presence of sleep apnea, such as:

- Apnea–hypopnea index (AHI) [13] is the number of times one has apnea or hypopnea during one night, divided by the hours of sleep. In other words, AHI score is the number of apnea and hypopnea events per hour of sleep. The severity of sleep apnea is determined based on the AHI score as follows: normal (AHI < 5), mild (5 ≤ AHI < 15), moderate (15 ≤ AHI < 30), and severe (AHI ≥ 30).
- Respiratory Disturbance Index (RDI) factor counts the number of times respiratory difficulties disturb one’s sleep. This includes, in addition to apneic and hypopneic events, respiratory effort-related arousals (RERA). RERA is the number of arousals from sleep resulting from increased respiratory effort. RDI is expressed as:
- RDI = (Number of apneas + Number of hypopneas + Number of RERAs)/sleep hours.

### 2.2. The Need for More Accessible Detection Mechanisms—Sensors to the Aid

While the PSG test is the gold standard in sleep apnea diagnosis, its availability, cost, requirement of trained staff, and limited capacity at sleep centers make it inaccessible to the common man, and sleep apnea is often undiagnosed or underdiagnosed, until the subject starts showing symptoms of long-term impact. Studies show that the percentage of elderly population in the world is increasing. Due to changing lifestyles, the number of elderly people living alone is also increasing. This has resulted in the emergence of geriatric healthcare homes, with round-the--the-clock staff support, albeit with high costs of maintenance. Technological advances in sensors, low power embedded systems, and machine learning have paved the way for more affordable and intelligent healthcare homes, with automatic monitoring of the subjects’ vital parameters [14]. One of the possibilities of such a system is the detection of sleep apnea.

Recent advances in sensing technologies have enabled the continuous collection of various vital parameters that can lead to monitoring sleep quality in multiple ways. The use of sensors to detect sleep apnea is a widely researched area, and the application of machine learning techniques to detect apneic conditions has been found to be accurate and reliable. The parameters used to detect sleep apnea, such as ECG and SPO2, their derivatives such as HRV, BCG, ODI, thoracic and abdominal signals, pressure, and sound [15], can be obtained from biomedical sensors, environmental sensors or vision-based systems.

- Biosensors allow sensing of vital parameters. For example, ECG sensors enable the detection of HRV and R-R intervals through signal analysis. They also enable the deduction of variations in QRS (Q wave, R wave, S wave) amplitude of ECG signals and ECG derived respiration. A variant of the ECG sensor, the single lead ECG sensor, is designed to be used with wearable devices. SPO2 sensors measure oxygen saturation levels in the blood. Barometric sensors measure blood pressure.
- Environmental sensors include those that can monitor the surroundings of the subject under study. For example, sound sensors allow nocturnal sound analysis by capturing snoring via microphones. Sounds and sound patterns during inhalation and exhalation will be different from normal when the upper respiratory tract is compromised. Inertial motion unit (IMU) sensors allow deriving the position of the sleeping subject. Sensors are also placed under the bed to enable non-intrusive monitoring.
- Vision based systems allow capturing of images through image and/or video feeds. Analysis of the images and video frames enables determination of the sleeping position of the subject under study.

Leelaarporn et al. [14] provide a comprehensive review of the utilization of sensors in four different areas of smart living, including sleep monitoring. Recent research trends in the area of sleep monitoring using several types of algorithms on pulse oximetry, ECG, sounds and respiration data are described in [16]. Flemons et al. [17] studies the utility of portable monitors in diagnosing sleep apnea in adults.

## 3. Machine Learning in Sleep Apnea Detection Based on Biomedical Markers in Wearable Devices

Machine learning applies mathematical modelling to detect or predict anomalies or patterns, to discover new knowledge from datasets. A model trained on a given dataset is used to classify new data. Machine learning can be supervised, unsupervised, or reinforcement learning [18]. Supervised learning algorithms take a labelled dataset as input and output a hypothesis that best fits the labelled dataset. A labelled dataset provides the algorithm with an outcome variable for each record in the dataset. Unsupervised learning algorithms do not have a labelled dataset for classifier training; rather, they detect patterns in the dataset to form clusters of similar records. Reinforcement learning has a feedback ingredient that incorporates reward points for records that get correctly classified, which substantiates classifier training. While there have been studies that uses spectral/waveform analysis of signals for sleep apnea detection [19,20,21], the ability of machine learning classifiers to learn from input datasets and generalize for future data makes it a reliable approach in this area of research. Most studies on sleep apnea detection rely on supervised learning.

The common set of parameters that is used to detect sleep apnea was explained in a previous section. Biomedical informaticians have used various machine learning techniques to predict the accuracy of sleep apnea diagnosis using these aforementioned parameters. Of late, the effectiveness of ensemble classifiers and deep learning techniques has also been investigated. The features used for sleep apnea detection could be reported directly from sensors, or extracted from various sensor observations. There has also been extensive research into utilizing observations from one or more of these sensors using data fusion to detect sleep disorders. Studies also include the impact of extracting statistical, time and frequency domain features from the parameters, and performing dimensionality reduction to downsize the feature vectors on the classifier performance. In the following sections, we look at how classic machine learning, deep learning, and sensor fusion techniques have been applied to detect sleep apnea. Deep learning can be considered as a specialized segment of machine learning; however, the manner in which feature engineering is accomplished differs greatly from each other. A snapshot of recent research on sleep apnea detection using machine learning and deep learning with biomedical sensors is presented in Table A1.

### 3.1. Classic Machine Learning Based Solutions

This section presents an overview of recent research in sleep apnea detection using classic machine learning techniques. In many research papers, single biomedical markers, such as SPO2, ECG, EOG, or EEG, have been used for the detection of sleep apnea. Among these, most studies focus on using SPO2 and ECG signals because of their correlation with apneic events—research shows that heart rate and systolic blood pressure increase in response to apneic events [22]. For example, in [12], SPO2 signals are used for OSA detection. During feature engineering, ODI, total time below saturation levels (tsa), and other six features were extracted from SPO2. Various variants of decision tree (DT) classifiers were used to obtain an accuracy of 93%. In [23] too, pulse oximeter parameters are used for sleep apnea detection. PPG measurements were obtained from SPO2 sensor and analyzed to derive heart rate and breathing effort information. The best classification performance of 87% was obtained when the Linear Discriminant Analysis was used on SPO2 features and the PPG features were combined. Another study that makes use of PPG measurements extracted from SPO2 readings is [24], in which statistical and time domain SPO2 and PPG features were extracted around SPO2 drops and averaged per patient. The impact of using SPO2 and PPG features on OSA detection was analyzed here. Three SPO2 based features and two PPG features were selected for training a support vector machine (SVM) classifier. Unlike [23], it was found that the classifier based on SPO2 features along with the subjects’ age yielded 77.7% accuracy, while the PPG features did not have any impact on the classifier performance. This research highlights that age is also a clear confounding parameter because of its correlation with cardiovascular health, and using age alone for OSA detection can yield a reasonable accuracy. In [25], four machine learning models are evaluated, to not just detect apnea but also ascertain its severity using only SPO2 information obtained at the patient’s home. A three-step process comprising feature extraction, feature selection, and classifier evaluation was conducted. A total of 16 features were extracted from SPO2 spanning statistical, spectral, and nonlinear domains, in addition to ODI, which were input to a Fast Correlation Based Filter feature selection algorithm. An AdaBoost model built with linear discriminants as base classifiers gave the best apnea severity classification accuracy. In [13], Mostafa et al. analyzes SPO2 signals from two public datasets using Deep Belief Network (DBN). The analysis shows that while the accuracy increases with the increasing number of hidden neurons, the increase is minimal, which may not justify the trade-off between classifier performance and processing requirements. Another study that detects sleep apnea conditions employs seven features and SVM [26]. This work not only detects but also corrects apneic events via a smart pillow. The setup consists of a wearable device with a pulse oximeter, a smartphone, and an adjustable pillow. The pulse oximeter on a wearable device senses the SPO2 signal and transmits it to a smart phone. The smartphone detects the SPO2 desaturation events and issues a pillow adjustment command. The adjustable pillow adjusts its shape and height according to the command. The adjustment effect is further monitored and evaluated by the pulse oximeter, providing a closed-loop feedback system between monitoring and corrective actions. Wrist band-mobile and mobile-pillow communication is over Bluetooth. A review of approaches for detecting sleep apnea specifically using pulse oximetry data is provided in [27].

ECG is another parameter that is commonly used in the detection of sleep apnea. Hassan et al. [28] compare various machine learning classifiers on a dataset generated by a single lead ECG sensor. Statistical moment-based and empirical mode decomposition features were extracted from the raw data. Post feature extraction, Naive Bayes, k-nearest neighbor (kNN), neural network, AdaBoost, Bagging, random forest, extreme learning machine (ELM), discriminant analysis (DA) and restricted Boltzmann machine were compared for performance. ELM gave the best accuracy of 83.77%. A dataset based on single-lead ECG was used in [29] as well to detect sleep apnea. In this study, segments of ECG signals were fed into dual-tree complex wavelet transform (DTCWT) to generate frequency sub-bands. Three statistical features—variance, skewness, and kurtosis—were extracted from the DTCWT output and analyzed to determine their suitability in detecting sleep apnea. LogitBoost gave an accuracy of 84.4%. Other classifiers analyzed include DA, kNN, Artificial Neural Network (ANN), ELM, SVM, AdaBoost and Bagging. ECG signals have also been used not just for the detection of sleep apnea, but also to determine its type [20].

Previous research indicates that parameters derived from ECG such as IHR, HRV, BCG, and CPC, have also been used as markers for training classifiers to detect sleep apnea. For example, certain studies [30,31] indicate that HRV measures have a great potential to boost OSA detection. Khandoker et al. [32] highlight the effectiveness of using HRV and EDR with an SVM classifier to attain 100% accuracy in the detection of apneic events. This study also uses SVM to estimate the relative severity of OSA. In [33], kNN, quadratic discriminant analysis (QDA) and SVM were applied on statistical measures of HRV. de Chazal et al. [34] use HRV, EDR, and CPC, obtained from single lead ECG signals, for sleep apnea detection. The analysis in this study shows that CPC features along with the time-domain-based HRV parameters gave the best classification performance, with an accuracy of 89.8%. The classifier algorithm used was multiple logistic discrimination. In [35], 24 time and frequency domain features are extracted from ECG signals. This included time domain features such as mean, median, standard deviation, and mode for each NN interval series, and frequency domain features such as normalized power in various frequency ranges, and the vegetative balance index. Feature selection was performed by discarding redundant features, leading to nine features being used for training decision trees, discriminant analysis, logistic regression, support vector machines, variation of kNN, and ensemble learning classifiers. Seo et al. [36] study sleep quality and stability assessment using sleep questionnaires and ECG. Respiratory and CPC parameters were extracted from ECG signals, and results found a significant correlation between AHI and CPC. Studies related to sleep analysis using EEG signals include [37,38].

### 3.2. Deep Learning Based Solutions

Deep learning techniques such as Deep Neural Network (DNN), Convolutional Neural Network (CNN), Recurrent Neural Network (RNN) and Long-Short Term Memory (LSTM) are being increasingly used for diagnosing sleep apnea, both on single markers as well as with sensor/feature fusion. Feature engineering and selection is crucial to the performance of intelligent solutions, especially in the biomedical domain [39]. One of the advantages of using deep learning is that they have the capability to learn relevant features from the raw data, using neurons, convolution and pooling layers. For example, Li et al. [40] argue that that while feature engineering is essential for improving the performance of classifiers, it often depends on human expertise which can tend to be subjective. In this study, unsupervised learning algorithms with sparse auto-encoders were used to learn features from ECG signals, to decouple the dependency of subjective human expertise on crucial feature engineering aspects. Classification was carried out using SVM and ANN, and the classification performance was refined using decision fusion and Hidden Markov Model (HMM). The accuracy obtained was 85% and the sensitivity was 88.9%. Another study that performs algorithmic extraction of features is [41]. In this, a single electrooculogram (EOG) signal was used to perform automatic sleep scoring. A three-layer DBN with 500, 200, and 100 neurons was used for feature extraction and label prediction. The predicted labels and original labels were used to train an HMM model. The average accuracy of the DBN–HMM model was 83.3%. This study attempts to establish that DBN can extract features by itself without manual intervention. Novák et al. [31] study how LSTM can enable the detection of temporal dependencies in features relevant to sleep apnea detection.

Wang et al. [42] use ECG signals for sleep apnea detection. R-R intervals and R-peak amplitudes were extracted from ECG signals, and time window ANN was applied for classification. The accuracy obtained was 87.3%. Mostafa et al. [13] describe a method to detect sleep apnea using SPO2 by calculating the AHI score. The deep learning algorithm used was DBN. Performance analysis was performed on two public datasets [43,44] with SPO2 values. Pathinarupothi et al. [15] detail the use of LSTM-RNN for the detection of sleep apnea severity and explores the relation between IHR and SPO2 towards this. The research shows that OSA severity detection can be solely based on either IHR or SPO2 signals.

In [45], IHR is used as the sole marker for sleep apnea detection. This paper argues that using only IHR and its derivatives can provide 85% accuracy at best, with simple classification algorithms for classifying minute-to-minute apnea. Therefore, LSTM–RNN was employed for the identification of sleep apnea and its severity. Various configurations of LSTM–RNN, post feature extraction and selection, were used for training, which yielded 99.99% accuracy in detecting sleep apnea. Erdenebayar et al. [22] describe a comparative study of the performance of deep learning classifiers on ECG signals—the classifiers are Deep Neural Network (DNN), 1D CNN, 2D CNN, RNN, LSTM and gated-recurrent unit model (GRU). The 1D CNN and GRU models were the best performing with an accuracy and recall of 99%. Other studies include [46,47,48].

### 3.3. Sensor/Feature Fusion Techniques

Extensive study has been performed to estimate the effectiveness of sensor or feature fusion techniques to detect sleep apnea. This involves the concurrent use of two or more parameters originating from different sources and performing classification based on the values of all these parameters. For example, Memis et al. [49] apply feature-level fusion of ECG and SPO2 signals. The temporal information from the ECG and SPO2 signals was fed as input to Naïve Bayes, kNN, and SVM classifiers. SVM gave the best accuracy of 96.64%. Xie et al. [50] also explores ensembles and data fusion over ECG and SPO2 signals. When analyzed separately, the research finds that SPO2 features can detect apneic episodes better than ECG features. Various classifier combinations trained on select features from SPO2 and ECG were then analyzed for performance. Feature extraction yielded 111 ECG and 39 SPO2 features, from which 8 ECG and 31 SPO2 features were selected for classifier training. The base classifiers were combined using maximum probability, average probability, product of probability and majority voting. Garde et al. [11] extract time-domain and frequency-domain features from SPO2 and PRV, and applies Logistic Regression to detect apnea/hypopnea events.

In [51], Prabha et al. make use of HRV and Respiratory Rate Variability (RRV) from ECG and respiratory effort signals (RES), respectively. A decision making system which fuses time-domain features from HRV and RRV signals, by combining their outputs with empirically calculated weights, produced an accuracy of 100%. The weight associated with time-domain HRV features was considerably higher than that of time-domain RRV features, which indicates that HRV has a higher correlation with sleep apnea detection than RRV, although the latter may be complementing the former. This analysis concludes that the time-domain features of HRV and RRV provide sufficient information to detect OSA. Other related studies include [52,53].

## 4. Other Solutions

In addition to devices that measure biomedical parameters, studies show the application of environmental sensors/devices such as microphones and cameras to ascertain the presence of sleep apnea. Literature also shows the application of health profiles to detect apnea and predict the AHI values to classify the severity of apneic events. Examples of such studies are summarized below.

### 4.1. Using Environmental Sensors

Sleep apnea detection can be performed with externally mounted devices or ambient sensors, other than biomedical sensors. One such technique for sleep apnea detection is based on smartphones. Camcı et al. [54] use sonar waves generated by smart phones, which give information about chest movements, to detect sleep apnea. The accuracy of the system was found to be dependent on the subject’s change of sleep position. Other techniques such as placing a microphone close to the subject’s nose and mouth were found to be obtrusive and impacting the sleep behavior of the subjects [55,56]. Another technique relies on the use of a 3D time-of-flight camera, which records the subject’s respiratory motion [57]. The signals pertaining to respiratory movement of abdominal muscles are analyzed to monitor sleep stages and detect apnea. Davidovich et al. [58] propose a novel algorithm for sleep apnea screening with a contact-free system based on a piezo-electric sensor. The setup consisted of a piezo-electric sensor, which recorded a combination of gross body motion, rib cage movements, and the cardioballistic effect. The specificity and sensitivity were found to be 89% and 88%, respectively.

Hafezi et al. [59] estimate sleep apnea severity from tracheal movements via an accelerometer attached to the participant’s suprasternal notch. 7 morphological features were extracted from tracheal movements, on which a deep learning classifier using a combination of CNN and LSTM, was applied. However, this method requires wearing a patch which may be inconvenient to the subjects.

In [60], Wang et al. propose a sleep breathing monitoring mattress which utilizes the ultra-wideband (UWB) physiological sensing technique. The UWB physiological sensing is accomplished via a series of very narrow and low power pulses over wideband. If apnea is detected, the head of the mattress is lifted up to increase blood oxygen saturation and ease the apneic condition. The methodology involved dataset collection using signals recorded from the experiment using Fast Fourier Transform (FFT), feature extraction using Principal Component Analysis (PCA) and classification using kNN, AdaBoost, DT, and SVM. kNN produced better results than the rest of the classifiers.

In [56], acoustic signals placed on the ceiling above the patient’s bed, were used. Subjects were classified into four sleep apnea severity groups according to their AHI. A two-stage filtering process to remove various unwanted noises and purify the sleep breathing sounds was applied. A total of 23 temporal and spectral features of the audio signal were extracted, which included the mel frequency, cepstral coefficients (MFCCs), spectral flux, and zero crossing rate. Logistic regression, SVM, DNN with 2 hidden layers were applied for classification.

In [61], machine learning models (kNN, AdaBoost, and DT) are applied on data generated by UWB sensors for sleep apnea detection. The experimental setup consists of a sleep breathing monitoring mattress which utilizes the UWB physiological sensing technique. The mattress also has a mechanism to lift up the head on detection of apneic events.

Avcı et al. [62] use abdominal, nasal, and chest respiratory signals and applied ensemble classifiers such as AdaBoost, random forest and random subspace to detect sleep apnea. Feature extraction and dimensionality reduction via PCA was performed to yield a best-case accuracy of 98.68%. Table A2 provides a snapshot of studies that apply machine learning to data generated by environmental sensors for sleep apnea detection. Ozdemir et al. In [63], a fully automatic apnea detection algorithm along with an early warning system to predict apneic events, is described. The algorithm also works on nasal respiratory airflow signals, on which feature extraction was performed. Subsequently, Randomly Select and Compute (RANSAC) algorithm was used for feature reduction on the original 39 features, and the set of features that is not significant for OSA detection is listed. SVM, kNN, and linear regression for classification are compared for learning and prediction of OSA episodes. The solution produced an accuracy of 87.6% of and sensitivity of 91.3%. Another study that makes use of airflow sensing signals for sleep apnea detection and classification of apnea severity is [55]. A total of 17 features from overnight airflow sensing samples were extracted, and fed into DNNs with various combinations of hidden layers and activation nodes per layer. The algorithm used the tanh activation function alongside the softmax classifier. Diagnosis of sleep apnea was performed using AHI threshold values of 5, 15, and 30 events/hour. The severity classification logic classified patients into four groups—no apnea, mild apnea, moderate apnea, and severe apnea. The best accuracy that DNN gave was 92.69%.

In [64], sleep data and 3D facial scans were used as features. The data collected was pre-processed for pose alignment and hole filling and analyzed using Matlab’s deep learning framework. The model thus generated was tuned for performance and used for classification. The accuracy reported was 69%. However, this method requires facial images of the subject, which restricts the subject’s degree of freedom while sleeping. Other studies in the area that use non-biomedical parameters include [65,66,67].

Non-wearable techniques for sleep apnea detection have certain advantages and disadvantages when compared with wearable devices. For example, wearable devices for sleep apnea detection have to be small in form factor and light-weight, while non-wearable techniques such as BCG-embedded beds or camera based systems do not have restrictions on their size or form factor. Another characteristic of comparison between wearable and non-wearable techniques is power consumption. Minimizing power consumption enables the wearable device to be on battery power for longer durations, which reduces the overhead of charging the devices. Power consumption of such devices occurs in three activities—sensing, processing, and communication. These three functions have to be optimized for energy saving to enable the device to be worn for long periods of time without recharging. In contrast, non-wearable devices can be connected to the main power supply, and hence need not be designed for optimized power consumption. One significant factor that affects the accuracy of sleep apnea detection in both techniques, is the placement of the sensors. Wearable devices allow round-the-clock monitoring of parameters since it does not restrict the parameter collection to a certain geographical region under study. However, non-wearable devices are sensitive to the sensing range of the devices. Environmental sensor-based systems also sometimes tend to be intrusive—for example, placing a microphone close to a subject’s face while sleeping could be uncomfortable for him/her. Camera-based systems may tend to be expensive and have higher power and bandwidth requirements. Due to all these aspects, wearable devices may be conducive to at-home sleep monitoring, while non-wearable techniques may be applied in hospital environments where the mobility of the subjects is more constrained.

### 4.2. Health Profiles for the Detection of Sleep Apnea

There has been research that highlights the significance of including a subject’s health profile in the diagnosis of sleep apnea and its severity. Mencar et al. [68] use 19 features including heart disease, diabetes, gender, BMI, age, smoking, hypertension and snoring, to explore methods to classify sleep apnea severity. Classification algorithms are applied to classify the severity of sleep apnea, and regression methods are applied to predict the AHI values. In another work, Ustun et al. [69] argue that medical information of subjects would be more suited to diagnose sleep apnea than real time sleep related symptoms. Features such as age, gender, BMI, presence of hypertension, history of heart failure, stroke, asthma, smoking, and snoring were used to train the classifiers. Seven classifiers including variants of Logistic regression, DT, and SVM were compared with a new machine learning model named SLIM (Supersparse Linear Integer Models). SLIM is a linear classification model for creating medical scoring systems, and this gave a sensitivity of 64.2% and specificity of 77%. The study supports the use of simple models with good generalization capabilities, especially for medical applications where datasets are prone to overfitting.

## 5. Discussion and Conclusions

In this study, we briefly summed up the causes and risks associated with sleep apnea, and the drawbacks of the related diagnostic processes. We outlined the parameters that help detect apneic events. Subsequently, we examined the application of machine learning in sleep apnea detection, with focus on wearable systems. We summarized the recent research that demonstrates feature engineering techniques and efficient use of classic machine learning, deep learning, and sensor/feature fusion algorithms to detect sleep apnea, and in some cases, classify its severity, using biomedical markers such as ECG, EEG and SPO2. The paper also briefly looked at the application of environmental sensors and information in subjects’ health profiles to ascertain the presence of sleep apnea.

From our analysis, an observation is that machine learning algorithms applied to datasets in the literature survey, produce varying degrees of accuracy. This indicates that the performance of the algorithms depends on various factors such as:

(i) Data collection modalities

Factors such as type of sensors, their placement, and frequency and sensitivity of measurements, affect the training of machine learning classifiers. Among the various biomedical parameters that aid in the detection of sleep apnea, we observe that the most common of them are those from ECG, SPO2, and EEG signals. The drawback of using ECG is that the signals generated by three leads or more require a resting ECG or an ECG Holter monitor, which may be restrictive for the subject under study because of the placement of leads. Single lead ECG can be embedded within wearable devices; however, the accuracy of such devices is less than those with multilead devices. Collection of EEG data also requires the subjects to wear a headgear while sleeping, which may cause inconvenience. SPO2 sensors, such as single lead ECG sensors, can be embedded within wearable devices and, in combination with the demographic information of subjects, has been proven to provide good results in the detection of sleep apnea. Environmental sensors may constrain the subjects to a certain area under observation while sleeping (such as bed-embedded BCG sensors). Some may introduce noise in the data collection, for example, acoustic sensors are prone to errors from ambient noise.

(ii) Dataset characteristics

Characteristics of data such as its distribution and dataset features, along with the pre-processing that has been applied to it also influences the efficiency of supervised training techniques. For a classifier to be well-trained, the dataset it trains on must be balanced. In the case of sleep apnea, it has to be ensured that the number of apneic events in the dataset are comparable with that of non-apneic events. In the absence of this, the classifier gets trained for the majority classes and misclassifies the minority classes. Additionally, appropriate data pre-processing techniques and feature engineering should be performed to fine tune the classifier training.

(iii) Labelling techniques

Training machine learning models for sleep apnea detection using supervised learning techniques, requires annotation of the records in the sleep dataset. Some of the standards used in sleep stage scoring from sleep study reports are the Rechtschaffen and Kales standard (R&K) [70] and American Academy of Sleep Medicine (AASM) [71]. In practice, apneic events are annotated manually by domain experts. The process involves correlation of the subject’s biomedical and physiological history with the sleep data, while adhering to the guidelines set forth by the standards. The dependency of annotation on the standards and subjective domain expertise may limit the generalization capability of the trained model.

The capability of a wearable device or an end-to-end system to store data for analysis, raise alarms on detection of abnormalities, and generate reports long-term is prudent, and especially useful in the context of geriatric care homes. Today, there are commercial devices that synchronize collected data to a smartphone periodically; however, a drawback of such a system is that at any given time, the device can be paired with only a single smartphone. The ability to support data collection and analysis at a central location would be especially beneficial in geriatric healthcare, where elderly people are saved the effort required to access and view their own reports.

## Data Availability

Not applicable.

## Appendix A

**Table A1:** Machine/deep learning based sleep apnea detection using biomedical sensors.

Reference	Year	Subject Demographics	Signal Used	Classifiers Applied	Feature Engineering Approach	Accuracy
[22]	2019	65 male, 21 female	ECG	DNN, 1D CNN, 2D CNN, RNN, LSTM, Gated recurrent unit	Performed by deep learning algorithms. For example, while using CNN, feature map was extracted using filter kernels by the convolution layer. Dimensionality reduction was performed by the pooling layer.	Accuracy: 99.0%
[40]	2018	Apnea-ECG @	ECG	DNN, HMM SVM, ANN	Sparse auto-encoders was used to learn features via unsupervised learning.	Accuracy: 85%;
[42]	2019	Apnea-ECG @	ECG	Time window ANN	R-R intervals and R-peak amplitudes were extracted from ECG signals. Further, 6 time domain and 6 frequency domain features from R-R interval, and 6 frequency domain features from R-peak amplitudes were extracted.	Accuracy: 87.3%
[34]	2016	Apnea-ECG @	ECG	Multiple logistic discrimination	Features extracted included RR-interval, EDR, CPC and their derivatives.	Accuracy: 89.8%
[31]	2008	Apnea-ECG @	ECG	LSTM, ANN and Elman Network	Feature extracted include: Time domain HRV parameters such as RMSSD (square root of mean squared differences of successive NN intervals), R-R mean (mean of R-R interval length) and NN50 (number of intervals longer than 50 ms) and frequency domain HRV features such as Low Frequency/High Frequency (LF/HF) ratio, total power for analyzed interval, Low Frequency (LF), High Frequency (HF), Very Low Frequency (VLF), normalized LF and normalized HF.	Accuracy: 82.1%
[33]	2010	5 male, 12 female, 26 years–67 years	ECG	kNN, QDA, SVM	Median, inter-quartile difference (75th and 25th percentile), and mean absolute deviations of the R-R intervals were computed for each epoch.	Accuracy: 90%
[28]	2015	Apnea-ECG @	ECG	Naive Bayes, kNN, ANN, AdaBoost, Bagging, Random Forest, ELM, DA, Restricted Boltzmann Machine	Statistical features such as mean, variance, skewness and kurtosis of the ECG signals were extracted.	Accuracy: 83.77%
[26]	2013	40 subjects	SPO2	SVM	For each detected SPO2 desaturation event, extract 7 features from a window of 150 s from the starting point of the SPO2 desaturation. Features extracted include no. of desaturation events, speed of decline in SPO2, in addition to statistical measures such as minimum and standard deviation of the SPO2 values.	Accuracy: 93.5%
[51]	2017	32 subjects, 18 to 75 years	ECG, RES	SVM	Time domain features (such as mean NN interval, standard deviation of NN interval, mean heart rate, RMSSD) and frequency domain (peak frequency, absolute power, relative power) features from HRV and RRV from ECG and RES, respectively, were computed.	Accuracy: 100%
[29]	2017	Apnea-ECG @	ECG	DA, kNN, ANN, ELM, SVM, AdaBoost, Bagging, LogitBoost	Skewness, variance and kurtosis were extracted and used for classifier training.	Accuracy: 84.4%
[49]	2017	Apnea-ECG @	ECG, SPO2	Naïve Bayes, kNN, SVM	Uses concatenation of temporal information from ECG and SPO2 signals.	Accuracy: 96.64%
[50]	2012	UCD #	ECG, SPO2	Adaboost, Decision Trees	Time domain features from SPO2, which measure the regularity, variability, and complexity of a time series, were extracted. From ECG, HRV and EDR-based features in both time and spectral domains were extracted.	Accuracy: 82%
[12]	2010	Apnea-ECG @	SPO2	Various variants of Decision tree classifiers	ODI indices from SPO2 were computed.	Accuracy: 93%
[15]	2017	Apnea-ECG @	ECG, SPO2	LSTM-RNN	Feature extraction was performed by LSTM.	Accuracy: 92.1%
[13]	2017	UCD #, Apnea-ECG @	SPO2	DBN	Feature extraction was by DBN.	Accuracies: 85.36% and 97.64%, respectively for the 2 datasets
[32]	2009	(1) Apnea-ECG @ (2) UCD # (3) 83 subjects; with mean +/− standard deviation age of 55.6 +/− 10.7 yrs	ECG	SVM	Feature extraction from HRV and EDR, using wavelet decomposition was performed. Feature selection was performed using a hill climbing algorithm. 14 HRV and 14 EDR were selected for classifier training.	Accuracy: 100%
[11]	2016	160 children (87 male, 59 female)	SPO2	Logistic Regression	Time and frequency domain features from SPO2 and pulse rate variability were used for classifier training.	-
[23]	2017	52 subjects	SPO2	Linear Discriminant Analysis	Features from SPO2 (such as number of desaturations > 3%, spread of SPO2, minimum and average of SPO2), PPG and PPG derived respiration were extracted for classifier training.	Accuracy: 87%
[35]	2020	Apnea-ECG @	ECG	Decision trees, DA, logistic regression, SVM, kNN, ensemble learning	24 time and frequency domain features were extracted. Feature selection by discarding redundant features, which resulted in a set of 9 features for classifier training	Accuracy: 98.7%
[52]	2020	Not specified	Respiration, SpO2, heartrate, 3-ACC signals	Gaussian Naïve-Bayes, ANN, kNN	Dataset was collected and labelled per AASM’s sleep apnea judgement criteria. A train:test ratio of 8:2 was used. 5-fold cross-validation was applied. The hyper-parameters of each machine learning algorithm were set by using the average of five cross-validation data sets.	Accuracy: 95%
[53]	2017	1983 subjects	Demographic information, EEG	Random Forest, XGBoost, and Light Gradient Boosting Machine	A total of 36 features were extracted from demographic information and EEG signals, including frequency and percentage of every sleep stage, time in bed, total sleep time, sleep efficiency and total number of one-step transitions overnight. Data imbalance was corrected using SMOTE analysis and feature selection was performed using statistical analysis.	Area under the curve: 0.9128
[46]	2019	SHHS (NSRR) %	Raw physiological respiratory signals	LSTM	LSTM was used to automatically learn and extract relevant features, and detect potential sleep apnea events. Direct respiration signals gave better accuracy than derived their signals such as EDR	Accuracy: 70% (approx.)
[47]	2020	Apnea-ECG @	ECG	Logistic Regression, SVM and 1D CNN	Time domain and frequency domain features of R-R interval were extracted for training logistic regression and SVM classifiers. There was no need for feature engineering with 1D CNN.	Accuracy: 88.23%
[48]	2020	MESA (NSRR) *	Respiratory signals	CNN, Markov Chain	Features learned by CNN.	Accuracy: 80.78%
[24]	2019	975 subjects	SPO2	SVM	Extracted features include simple time-domain (e.g., amplitude and length of desaturation), statistical (e.g., minimum and mean SpO2 value) and desaturation severity (e.g., area below SpO2 baseline) and quasi periodicity features (e.g., phase rectified signal averaging (PRSA)).	Accuracy: 77.7%
[41]	2015	SleepEDF Database [Expanded] ~	EOG	DBN	Feature extraction was performed by DBN.	Accuracy: 83.3%
[25]	2019	320 subjects, age 54.8 +/− 13.5 years	SPO2	Linear Discriminant Analysis, Logistic regression, Bayesian Multi Layer Perceptron, AdaBoost	Time domain features that characterize central tendency, dispersion, asymmetry, and peakedness of a given time series, frequency domain features such as PSD of SPO2 signals, ODI3 and non-linear measures were computed.	
[45]	2017	Apnea-ECG @	ECG	LSTM-RNN	Continuous time series IHR measurements were converted to a series of feature vectors, for each beat window.	Accuracy: 99.99%

@ Apnea-ECG: 70 ECG signal recordings extracted from PSG recordings with a 16-bit resolution, a sampling rate of 100 Hz [43,72]. # St. Vincent’s University Hospital/University College Dublin (UCD) Sleep Apnea Database: 21 males, 4 females; age 28 years—68 years [44]. % SHHS dataset Sleep Heart Health Study: The dataset consists of 5804 adults of age 40 and older. A subset consisting of 1008 female and 1092 male patients with mean age 62.5 ± 12.6 (standard deviation) years was used in the study cited [73]. * MESA (NSRR): 6814 subjects; age 45 years–84 years [74]. ~ SleepEDF Database [Expanded] 20 subjects; age 25 year–34 years [75].

**Table A2:** Machine/deep learning based sleep apnea detection using environmental sensors.

Reference	Year	Subject Demographics	Signal Used	Classification Approach	Methodology	Accuracy
[59]	2020	Ages 18–85 years	Tracheal movements	CNN+LSTM	Recorded tracheal movements were filtered using a bandpass filter with cut-off frequencies of 0.1 Hz and 25 Hz. Time-series sliding windowing technique with a window size of 10 s was applied. 21 morphological features were extracted from each window.	Accuracy: 84%
[64]	2018	39 male, 30 female adults	Facial images	CNN	Involved data collection, pre-processing, model generation and tuning, and classification, using Matlab based deep learning framework	Accuracy: 69%
[60]	2019	5 subjects simulating sleep apnea conditions	UWB signals	AdBoost, Decision tree, SVM, kNN		Accuracy: 98%
[54]	2017	4 subjects simulating sleep apnea conditions	Accelerometer and sonar waves from smart phones	kNN, CART	Mean, variance and range for accelerometer data and the noise level were extracted, and subsequently, no. of breaths per minute was calculated.	Accuracy: 97.7%
[58]	2016	77 male, 19 female 23–88 years	Piezo-electric sensor	Signal analysis	Gross body motion, rib cage movements, and cardioballistic effect was recorded by the piezoelectric sensor. Time and frequency domain features were extracted from motion, respiratory rate and inter-beat intervals, to calculate AHI.	-
[56]	2018	120 subjects, including 3 children and 4 adolescents	Acoustic signals from microphone placed at a distance of 1.7 m above the subject’s bed	Logistic regression, SVM, DNN with 2 hidden layers	Several temporal and spectral characteristics of audio signals such as the mel frequency cepstral coefficients (MFCCs), spectral flux, and zero crossing rate were extracted.	Accuracy: 92.5%
[55]	2018	MrOS [TBD]	Airflow signals from a thermistor placed in front of the nose	SVM, AdaBoost, Regression, Deep Neural Networks	Seventeen time domain features were extracted from airflow signals, after subsampling and filtering.	Accuracy: 92.69%
[63]	2016	6 subjects	Nasal respiratory airflow signals	SVM, kNN, Linear Regression for Classfication	15 time-series features of OSA periods such as mean, variance, minimum, maximum, median values of signals were extracted. Minimum, maximum, average inspiration/expiration amplitudes and durations of nasal airflow signal were also extracted. Feature reduction was performed using RANSAC algorithm.	Accuracy: 87.6%
[62]	2015	Apnea-ECG	Abdominal, chest and nasal respiratory signals	AdaBoost, Random Forest and Random Subspace	Wavelet transform based on feature extraction methods are applied on 1 min length respiration signals.	Accuracy: 98.68%
[61]	2015	3 male, 1 female Age 48 ± 6.9 years	Reflect pulses from Impulse Radio Ultra-Wide Band (IR-UWB) radar panel	Linear Discriminant	Normal and apnea epochs were extracted from the IR-UWB data. 15 statistical features were derived from these extracted epochs.	Accuracy: 73%
[65]	2020	9 subjects Age 65 years or more	Signals from pressure sensitive mat	Temporal convolutional network (TCN), bidirectional LSTM	Data pre-processing included occupancy extraction, bandpass filtering, signal combination, concatenation and normalization. TCN and bidirectional LSTM approaches were compared with SVM and threshold based approaches.	Accuracy: 95.1%
[66]	2020	4 male, 4 female Age 25 years–55 years	Respiratory signals from accelerometer and pressure transducer	CNN	Sliding window approach was used for signal processing. Proposes a system of continuous monitoring of breath, from an accelerometer-based device worn around the subject.	Accuracy: 88%
[67]	2019	20 adults	Speech signals	Random Forest	Offline detection of OSA using speech/voice analysis. This is based on the fact that speech properties of OSA patients are altered. Feature extraction was performed on audio files using Random Forest feature selection and Mann–Whitney U test ranking.	Accuracy: 87.5%
